# *Plants:* An International Scientific Open Access Journal to Publish All Facets of Plants, Their Functions and Interactions with the Environment and Other Living Organisms

**DOI:** 10.3390/plants1010001

**Published:** 2012-02-06

**Authors:** W.G. Dilantha Fernando

**Affiliations:** *Founding Editor-in-Chief of Plants*, Department of Plant Science, University of Manitoba, Winnipeg, Manitoba, R3T 2N2, Canada; Email: D_Fernando@umanitoba.ca

**Keywords:** n/a

## Abstract

Plants are one of the two major groups of living organisms that are an essential entity to the function of the biosphere. Plants can be found in all known parts of the earth, in all shapes and sizes. They include the green algae, mosses, ferns, vines, grasses, bushes, herbs, flowering plants and trees. Although some plants are parasitic, most produce their own food through photosynthesis. Most plants initiate from a seed. The importance of plants in the food chain dates back to ancient times. The first humans gathered wild plants for food. As settlements developed, food crops were cultivated, leading to selection of high-yielding cultivated varieties to feed the growing populations. Unlike plants, humans and other animals are unable to manufacture their own food. Therefore, they are dependent, directly or indirectly, on plants. Plants are found in natural ecosystems such as rain forests, and also in agricultural areas and urbanized settings. They are an essential part of our daily lives providing food, clean air, and important ecosystem functions. The study of plants and their function could be considered the most complex of interactions. From the time a seed germinates, it goes through a myriad of physiological processes that can be closely studied using modern tools and molecular biological methods. An open access journal such as *Plants* will give millions of readers access to that information around the world.

## Introduction

I would like to put plants in different areas of importance to address their usefulness to the world.

## Plants as a Source of Food:

Plants are one of the two major kingdoms of life forms. They are the only life forms that can produce their own food using energy from sunlight. Plants have green pigment called chlorophyll in their cells, mainly in the leaves. This pigment allows plants to make food from sunlight, water and carbon dioxide in a process called photosynthesis. Plants manufacture much more food than they can readily utilize and they store up this excess as a reserve in leaves, stems, roots, fruits or seeds, for future use. It is this supply of reserves that is used by humans and animals. Although many synthetic chemicals can replace other plant-derived materials, there is no substitute for plant-derived food. Without plants, life would not be sustained on earth. People depend upon plants to satisfy their basic human needs such as food, clothing, shelter, and medicine. To date, these basic human needs are growing rapidly because of a growing world population, increasing incomes, and urbanization. Plants make up the largest proportion in our diet, in many countries the staple diet comes from rice or wheat. Humans get 85% of their calories from 20 plant species and interestingly 60% of that comes from three plant species, wheat, rice and maize [1].

The essential foods produced by plants are carbohydrates, fats and proteins, each being of value in its own way to human and animal metabolism. There are also mineral salts, organic acids, vitamins and enzymes that are required for general health. Plants have different parts that are used as food. The most important of these for humans are seeds and fruits, which are found in cereals and small grains, legumes and nuts. These contain large amounts of nutritive material and have proportionately low water content, which enhances their value because they can be stored and transported with ease. Next in importance as sources of food are roots, tubers, bulbs and other vegetables from the soil. Their value is less because they contain more water. The leafy parts of plants contain comparatively little stored food but they are necessary because of the vitamins and mineral salts they contain and the mechanical effect of their indigestible cellulose. This is true also of the fleshy fruits that may also contain various organic acids.

## The Environmental Impact:

Since the development of agriculture 10,000 years ago, plants have been viewed primarily as a source of food. However their impact and role is far greater and far older. The emergence of photosynthetic plant life as a dominant force on earth transformed our atmosphere into the oxygen-rich air we breathe. In addition to releasing oxygen, plants use carbon dioxide to complete the carbon cycle and recycle the CO_2_ released by humans and other heterotrophs. CO_2_ uptake can also help mitigate the greenhouse effect and climate change. Therefore plants are very important to maintain the balance in an ecosystem and drive most of important biological processes.

Plants are integral to the ecosystems they inhabit and contribute to enriching their environment. Plants improve their habitat by constantly filtering the air, water, and soil they reside in. Phytoremediation is the process of removing pollutants by either containing, degrading, or eliminating contaminates such as solvents, pesticides, metals, crude oil and its derivatives. Plants also actively shape their environment by creating local climates in forests and marshes and reduce the risk of natural disasters such as droughts by retaining ground water. Conversely plant roots minimize soil erosion by rain and wind by holding the top soil in place and controlling the flow of water.

However unlike plants, humans do not always contribute positively to their environment or use their resources effectively. Whereas plants foster diverse ecosystems, humans have caused extinction and promoted artificial selection creating a myriad of new problems. Of the more than 300,000 species of plants, less than 2% have been tested for medical potential while over a quarter of all modern drugs are derived from plants. While we imagine food scarcity to be the problem, the World Health Organization (WHO) reports that 1.5 billion people are overweight and more people die from obesity than starvation. While there is ample food to feed everyone, localized social inequality, political unrest, and economic policies drive hunger. Many of these solutions can be adapted to human problems. A sustainable outlook would necessitate accepting our role as a dependent subsystem of the ecosphere and from there striving to maintain our habitat.

Throughout the world, plants are extremely important ecologically and are found everywhere. It could be stated that plants have essentially led to the beginning of aerobic life due to their production of oxygen required for life to form. It is for this reason that precautionary measures must be taken in order to sustain adequate plant populations necessary for our survival.

Plants are also important in the regulation of global climate change. Carbon dioxide is consistently being dispersed into the surrounding atmosphere, creating conditions where temperatures become warmer. Trees are effective in removing the carbon dioxide from the surrounding air and transforming it, by photosynthesis into oxygen, therefore in some aspect controlling the temperature of the atmosphere. Light emitted from the sun also contributes to the heating of the atmosphere. Where plants are present on the earth’s surface, the solar radiation is mostly absorbed by the plants, reducing the amount of heat reflected into the surrounding air.

## Biodiversity:

Plants are important environmentally for various reasons. To some animals, one plant species may be their only source of food, and if taken away, the animal depending on this species may not survive. For this reason, we must try to preserve natural habitats containing native vegetation from which these animals feed. The removal of one plant species may be linked to the removal of one animal species which could also be detrimental in removing an entire ecosystem of living entities. Certain animals consider the plants that they live among as their home. For example, by replacing a forest with agricultural land, we are essentially removing the habitat of larger species such as bears, deer, owls, *etc*. These modifications may also lead to the extinction of various animal species. Preserving the biodiversity of plant species allows us to also sustain a population of various animal species within their ecosystem. Symbiotic relationships are also created among trees and other living entities (fungi, bacteria, *etc*.) benefiting both the tree and the entity. As an example, plants require a sufficient amount of water from the soil and therefore allow fungi to attach to the roots to gain additional moisture deeper into the soil, while the fungi benefit from the nutrients provided by the plant. A diversity of organisms living within an ecosystem allows for sufficient interaction among life forms.

Biodiversity of plants on the Earth provides aesthetic value to humans, especially the flowers that make our gardens so pleasant. Trees provide wood and many other useful possessions. Plants, especially trees can help make and preserve soil. Researchers have found that plant biodiversity can aid in reducing the effects of climate change [2]. In recent years, several plant species are attaining endangered species status, and the biodiversity on Earth is threatened. According to a 2010 study conducted by the Royal Botanic Gardens in the UK, 22% of the approximately 380,000 known plant species (or about 83,600 plant species) are endangered [3]. It is more important than ever to turn around the loss of biodiversity and conserve all plants.

Due to the advanced techniques of molecular biology and genomics, we can study their function in an environment and understand plants better. However, with these advances, the nature of plant relationships and classifications of some species is constantly changing, which is one vital area we could deal with and learn from journals such as *Plants*.

This brings me to why an open access journal on plants at this juncture is so important. An open access journal serves three main purposes. Firstly, it attracts and publishes high quality peer-reviewed publications; secondly, allows free access to the scientific community to read these articles at any time; and thirdly allows free access to the general public (including school children and university students) to read articles of interest to them. Understanding plants through a scholarly journal that attracts research publications in a variety of areas will help the reader appreciate the value of plants and their sustainability in their ecosystem. Although several journals cover topics related to plants and their function and interactions with other organisms, this open access journal would be a platform to bring all plants and related disciplines under one journal. The on-line open access format will help and strengthen research communication between researchers and countries. It will also immensely help the scientific community in third world countries who cannot afford to subscribe to other plant related journals, to have access to some novel research published in our open access journal. The main aim of our journal is to encourage scientists and research groups to publish theoretical and experimental results of research in all fundamental and applied fields of plant science.

The publication costs will be waived for all contributors in 2012. I encourage all authors to take advantage of this generous offer and publish your research articles in *Plants*. Each article will be subjected to a rigorous peer review. The peer review process is led by carefully selected world-renowned scientists from various disciplines that have broad expertise covering most aspects of plants. Once accepted, your article will be published immediately giving access to millions of readers around the world. Our wish is that you will select *Plants* as your foremost journal to publish high quality, cutting edge research. *Plants* will entertain original research articles, mini reviews, reviews, communications, short notes and suggestions for special and focused issues in the fields of structural, functional and experimental botany. In addition to fundamental disciplines such as morphology, systematics, physiology and ecology of plants, the journal welcomes all types of articles in the field of applied plant science. We encourage your active participation by contributing articles to *Plants* in the near future.
